# Stress-Related Gene Expression in Liver Tissues from Laying Hens Housed in Conventional Cage and Cage-Free Systems in the Tropics

**DOI:** 10.1155/2024/4107326

**Published:** 2024-01-13

**Authors:** María Paula Herrera-Sánchez, Roy Rodríguez-Hernández, Iang Schroniltgen Rondón-Barragán

**Affiliations:** ^1^Poultry Research Group, Laboratory of Immunology and Molecular Biology, Faculty of Veterinary Medicine and Zootechnics, Universidad del Tolima, Altos de Santa Helena, Postal Code 730006299, Ibagué, Tolima, Colombia; ^2^Immunobiology and Pathogenesis Research Group, Laboratory of Immunology and Molecular Biology, Faculty of Veterinary Medicine and Zootechnics, Universidad del Tolima, Altos de Santa Helena, Postal Code 730006299, Ibagué, Tolima, Colombia

## Abstract

Global egg production is mainly based on cage systems, which have been associated with negative effects on the welfare of birds. Stress factors in restrictive production systems can lead to changes in gene transcription and protein synthesis, ultimately impacting the quality of poultry products. The liver serves various metabolic functions, such as glycogen storage, and plays a crucial role in animals' adaptation to environmental changes. Consequently, both internal and external conditions can influence liver functions. The aim of this study was to evaluate the gene expression of *AGP*, *CRP*, *NOX4*, *SOD1*, *CAT*, *GPX1*, *SREBF1*, and *FXR* in the liver of laying hens under two different production systems. Liver tissues from Hy-Line Brown hens housed in conventional cage and cage-free egg production systems at 60 and 80 weeks of production were used. mRNA transcript levels were determined by qPCR using the relative quantification method and *ACTB* as the reference gene. *AGP*, *SOD1*, and *SREBF1* gene expressions were significantly higher in the conventional cage group at the 60 weeks of production. Furthermore, the mRNA levels of transcripts related to oxidative stress and lipid metabolism were higher in the group of laying hens housed in conventional cages compared to those in cage-free systems. These results suggest differential gene expression of genes related to oxidative stress in liver tissues from hens housed in conventional cages compared to cage-free systems. The conditions of the egg production system can impact the gene expression of oxidative stress and lipid synthesis genes, potentially leading to changes in the metabolism and performance of hens, including egg quality.

## 1. Introduction

Global food production demand has led to the development of egg production systems focused on enhancing productivity parameters through genetic selection and intensive production [1, 2]. However, pressure from egg consumers is demanding a change from intensive systems and adopting alternatives to the conventional cage house systems in accordance with animal welfare [3]. Nowadays, free-range production systems, according to consumer perception, produce tastier and healthier eggs than those produced in cage productions [4]. In the European Union, the conventional cage production system has been prohibited due to its low welfare standards and the limitations of the animals to perform natural behaviors [5, 6]. Nonetheless, conventional cages remain the predominant system in countries such as China, Brazil, Japan, Mexico, Turkey, and Russia [7].

The inability to perform natural behavior and the higher stocking density (SD) in conventional cage cause chronic stress in the hens and ducks [8, 9]. High SD in cages restricts access to food and water, leading to an increase in injuries and diseases among some birds [10]. Furthermore, the stress response triggers the activation of the hypothalamic-pituitary-adrenal axis (HPA), resulting in the release of glucocorticoids (GCs) that alter the physiological state and immunological response, with deleterious effects on the liver [11]. Additionally, elevated GC levels can impact digestion, energy, and triglyceride metabolism, activate lipogenic genes leading to hepatic steatosis, and influence fatty acid metabolism [8, 12, 13].

The acute phase proteins (APPs) are produced by the liver in response to infections and inflammatory stimuli [14]. The APPs gene expression has been associated with stress as part of the general physiological response mediated by the HPA axis, and in chickens and turkeys, they have been utilized as stress biomarkers [15–17]. Furthermore, stress factors lead to heightened metabolism due to elevated levels of GC, resulting in the increased production of reactive oxygen species (ROS) that cause long-term damage to DNA, lipids, and proteins as a consequence of oxidative stress and also impact bile acid biosynthesis [12, 18, 19]. Additionally, the high stocking density (SD) in conventional cages affects hens by increasing cholesterol and triglycerides in the plasma, and this effect may be mediated by the upregulation of the sterol regulatory element-binding transcription factor 1 protein (*SREBF1*) gene [20, 21]. Consequently, the aim of this study was to evaluate the transcripts of *AGP*(alpha-1-acid glycoprotein), *CRP*(C-reactive protein), *NOX4*(NADPH oxidase 4), *SOD1*(superoxide dismutase 1), *CAT*(catalase), *GPX1*(glutathione peroxidase-1), *SREBF1*(sterol regulatory element-binding transcription factor 1), and *FXR*(farnesoid X receptor) genes in the liver tissue from hens housed in conventional-cage and cage-free systems.

## 2. Materials and Methods

### 2.1. Ethical Approval

All procedures were approved in Act 007-2020 issued by the Bioethics Committee of the University of Tolima according to the Colombia Laws.

### 2.2. Study Population

The tissue samples were obtained from a previous research of the Poultry Research Group of the University of Tolima [22]. Briefly, under commercial conditions, 60,000 one-day Hy-Line Brown pullets were placed in cages (mod manure belt brood grown) with an area of 76.22 × 66.05 m and a density of 16 pullets/cage (314.645 cm^2^/bird). Pullets were reared with the same sanitary conditions, management, and feed program until 15 weeks (wk) of age. Later, the same birds were transferred into two different housing systems, conventional-cage (CC) and cage-free (CF), on the same farm up to 82 wk of age. A total of 45,000 hens were housed in a CC system in a pyramidal multideck battery of vertical cages in Californian type facilities (40 × 40 × 40 cm). Each battery had four stages and nipple drinkers, and the house had a cooling panel ventilation system. For this study, 720 hens were evaluated in 15 replicates of 12 cages each (48 birds/replica) for a total of 180 cages assessed in the CC system. The CF system evaluated was an aviary type; it had a floor-deep litter using rice husks in conventional houses, open sheds (mesh-sided), and natural ventilation (wind only) with perches and community nests, without access to grass or insects. A total of 14,850 hens (1,111 cm^2^/bird) at 16 wk of age were distributed in the CF system. Two poultry houses were divided into fifteen replicates (rooms) with 990 hens/room (9 hen/m^2^). Diets were formulated based on the Hy-Line Brown layer management guidelines and fed the same diet in both systems, and a lighting program of 14L:10D was used [22].

### 2.3. Samples, RNA Extraction, cDNA Synthesis, and Endpoint PCR

The sample collection was performed at 60 and 80 wk of hen's age. Three hens (*n* = 3) at 60 wk and six hens (*n* = 6) at 80 wk per production systems were randomly selected from different replicates and euthanized by cervical dislocation followed immediately by decapitation [22]. Approximately 0.5 g of liver tissues was collected from the hens sampled and stored in RNAlater® stabilization solution (Thermo Scientific, Wilmington, DE, USA). Total RNA was extracted from 0.5 g of liver tissues using RNA-Solv Reagent (OMEGA, Norcross, GA, USA) and the concentration was measured using the NanoDrop One (Thermo Scientific, Wilmington, DE, USA). cDNA was synthesized with the GoScript™ Reverse Transcription System kit (Promega, Madison, WI, USA), following the manufacturer's instructions.

End-point PCR was used to determine cDNA quality through the *ACTB* gene amplification. The reaction was performed using GoTaq® Flexi DNA polymerase (Promega, Madison, WI, USA) with a total volume of 25 *μ*L:14.8 *μ*L of distilled deionized water, 5 *μ*L of 5X green GoTaq® Flexi Buffer, 1 *μ*L of dNTPs (1.5 mM) (Invitrogen, Carlsbad, CA, USA), 1 *μ*L of each specific primers for each gene (forward and reverse) (10 pmol/*μ*L) (Table 1), 1 *μ*L MgCl_2_ (25 mM), 0.125 *μ*L of GoTaq® Flexi DNA polymerase (Promega, Madison, WI, USA), and 1 *μ*L of cDNA as template. In a ProFlex PCR System (Applied Biosystems, Carlsbad, CA, USA), the amplification was carried out and the thermal profile was as follows: denaturation step at 95°C for 3 min, 35 cycles of denaturation at 95°C for 30 s, annealing at 55°C for 30 s, extension at 72°C for 30 s, and final extension at 72°C for 5 min. The electrophoresis was performed using 2% agarose gel with HydraGreen™ as DNA dye (ACTGene, Piscataway, NJ, USA) in the PowerPac™ HC (Bio-Rad, Hercules, CA, USA).

### 2.4. Quantitative Polymerase Chain Reaction (qPCR)

The expression of genes of interest (Table 1) was measured using the Luna® Universal qPCR Master Mix (New England BioLabs Inc., Beverly, MA, USA) in a QuantStudio 3 Real-Time PCR System (Thermo Fisher Scientific, Waltham, MA, USA), by fast ramp program, according to the manufacturer guidelines. The thermal cycling conditions were denaturation for 1 min at 95°C, 40 cycles of denaturation for 15 s at 95°C, and annealing of 30 s at 60°C. Subsequently, a melting step was performed at 95°C for 1 s and 60°C for 20 s, and a continuous rise in temperature to 95°C at a rate of 0.15°C per second. Each sample was run in duplicate. The relative gene expression was determined by the 2^−ΔΔCt^ method [23], expressed as fold change, and the *ACTB* (actin beta) gene was used as the reference gene [24].

### 2.5. Statistical Analysis

The data were analyzed by descriptive analysis and the Shapiro–Wilk test. Additionally, the difference in gene expression was assessed using a *t*-test or the Mann–Whitney test, according to the normal distribution of the data, and was expressed as the mean ± SEM. The analyses were performed using GraphPad Prism v 10.0 (La Jolla, USA), and the statistically significant differences were considered at *p* < 0.05.

## 3. Results

### 3.1. Gene Expression of Acute Phase Proteins' Genes

**Figure 1:**
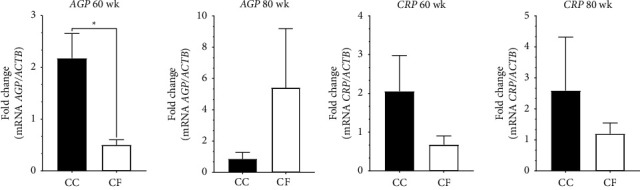
Relative acute phase proteins gene expression in the liver tissue of laying hens at 60 wk and 80 wk of age. (a) *AGP* mRNA levels and (b) *CRP* mRNA levels; CC: conventional cage production system and CF: cage-free production system. The actin beta (*ACTB*) gene was used as a reference gene. ^*∗*^*p* < 0.05.

The gene expression of *AGP* in the liver was significantly higher in the CC than in the CF egg production system at 60 wk (*p* = 0.026) (Figure 1). Additionally, the mRNA levels of the *AGP* gene at 80 wk of production showed a higher level in the CF group than in the CC group. Finally, the expression of *CRP* transcripts has a tendency for higher expression values in the liver from the CC hens at 60 and 80 wk of age (Figure 1).

### 3.2. Gene Expression of Oxidative Stress Genes

**Figure 2:**
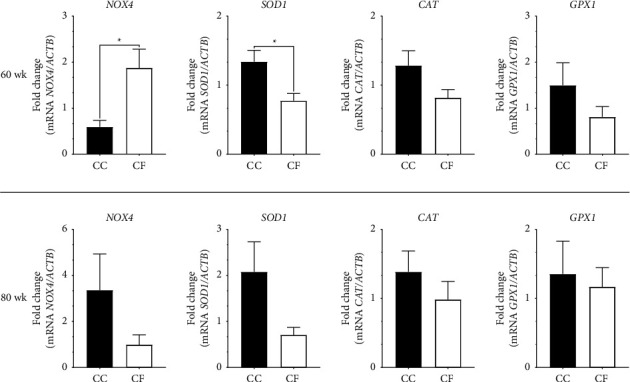
Relative oxidative stress gene expression in the liver tissues from laying hens at 60 wk and 80 wk of age. CC: conventional cage production system; CF: cage-free production system. The actin beta (*ACTB*) gene was used as a reference gene. ^*∗*^*p* < 0.05.

At 60 wk, the *NOX4* mRNA levels were higher in liver tissues from hens in the CF group than in the CC group (*p* = 0.04), in contrast to the *SOD1* transcripts levels, which were significantly higher in the CC group than in the CF group (*p* = 0.045) (Figure 2). The *CAT* and *GPX1* did not exhibit statistical differences; however, the mRNA levels showed a trend of higher values in the CC group. There were no significant differences in the *NOX4*, *SOD1*, *CAT*, and *GPX1* transcripts at the 80 wk; nevertheless, the transcript levels of these genes showed higher numerical values in the CC group compared to those of the CF group (Figure 2).

### 3.3. Gene Expression of Lipid Metabolism Genes

**Figure 3:**
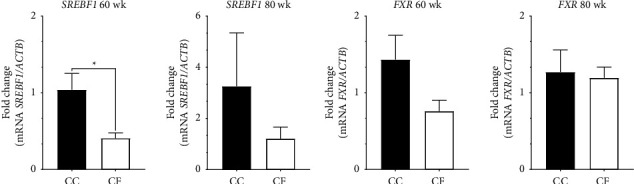
Relative gene expression in the liver tissue of laying hens at 60 wk and 80 wk of age. (a) *SREBF1* mRNA levels and (b) *FXR* mRNA levels; CC: conventional cage production system and CF: cage-free production system. The actin beta (*ACTB*) gene was used as a reference gene. ^*∗*^*p* < 0.05.

*SREBF1* transcripts were significantly upregulated in the liver tissues from hens housed in the CC group at 60 wk compared to the CF group (*p* = 0.047). At 80 wk, mRNA levels of the *SREBF1* in the CC group showed higher numerical values than those in the CF group; however, there were no significant statistical differences. The *FXR* mRNA levels, despite showing no statistical differences, CC group exhibited higher mRNA levels than the CF group, indicating a potential impact of CC production on *FXR* gene expression during the two weeks evaluated (Figure 3).

## 4. Discussion

The CC system focuses on maintaining a high stocking density (SD), i.e., a higher number of hens per area unit, to improve economic profits for the producer [25]. However, this approach has a detrimental impact on the physiological response to stress, ultimately affecting productive performance [26]. Stress refers to the imbalance of homeostasis caused by external or internal factors that alter physiological conditions through a neuroendocrine response [27]. In laying hens exposed to stress, egg production could be affected by the liver response which modulates the metabolism to maintain homeostasis [28]. In addition, the higher SD has been associated with increased mortality and disturbances, primarily due to heat stress, impacting the liver through oxidative stress and inflammation [29–31]. Several studies have suggested that stress can be induced by densities lower than 465 cm^2^/hen, whereas in our study, the density was 450 cm^2^/hen in CC systems [25, 32]. Currently, animal welfare measurement has been focused on behavioral identification and stress measurements using corticosterone and heterophils/lymphocyte ratio, among others; however, molecular biology techniques can be helpful to estimate the physiological and biochemical responses using specific biomarkers to monitor animal welfare such as heat shock proteins (HSPs) and acute phase proteins (APPs) [33]. Indeed, it is necessary to incorporate stress-related genes in poultry production to assess the effect of the production system and establish management solutions to mitigate the negative impact on egg production [34].

APPs increase their levels in response to stress, infection, trauma, and inflammation [14]. They are used as stress biomarkers, as noninfectious factors such as heat and transport can modulate the immune system and the release of APPs, enabling the assessment of animal health and welfare [35]. Alpha-1-acid glycoprotein (AGP) is a moderate positive APP, with concentrations increasing 2–10-fold during the acute phase response [36, 37]. The function of the AGP is to inhibit the activation of neutrophils to avoid inflammation and in macrophages to increase the IL-1 receptor secretion [38]. In stress, liver functions can be influenced by glucocorticoids (GCs), with cortisol inducing the overexpression of the *AGP* gene [16]. Other corticosteroids, such as corticosterone (CORT), are used as biomarkers of acute and chronic stresses in birds and have shown higher plasma levels in hens housed in CC systems [9, 39]. In our study, the gene expression of *AGP* in the liver was significantly higher in the group of hens housed in a CC than in the group of the CF system at 60 wk of production. Related results were reported in broiler chickens where serum *AGP* levels were higher in heat-stressed chickens compared to the control group [40]. Additionally, in embryos of chicks exposed to higher temperatures, *AGP* levels increased throughout the incubation days due to heat stress [41]. Furthermore, Zulkifli et al. [15] indicated that laying hens administered with CORT had significantly higher serum levels of *AGP* after seven days of injection. Therefore, the upregulation of *AGP* expression in laying hens housed in CC systems may be related to the stress caused by the production system conditions, possibly mediated through higher levels of CORT.

Meanwhile, the levels of *AGP* mRNA at 80 weeks in hens housed in CF showed higher levels in the CF group compared to the CC group. Previously, it was mentioned that the animals were healthy with no evidence of clinical signs that could indicate that the overexpression of the *AGP* in the CF group is related to other factors. Salamano et al. [42] reported that when commercial laying hens of the ISA Brown variety were accommodated in a free-range system, the AGP serum levels increased over time at 15 days, two months, and four months. This variety of laying hens is adapted to cage systems and shows less adaptability to other environments and systems [42]. In our study was used Hy-Line Brown breed, which showed an increase in *AGP* mRNA levels from 60 weeks to 80 weeks. According to this, *AGP* behavior may be related to the fact that commercial breeds such as Hy-Line Brown have lower adaptability, which depends on the genotype and this breed tends to develop fear in cage-free systems compared to native-breed hens [43]. The results of genetic selection for better productive characteristics are made under stable conditions, and subjecting the birds to a variable environment, such as cage-free systems, can generate stress and fear [43, 44]. Following the APPs, the C-reactive protein (CRP) is a positive APP that increases its levels under a stimulus that according to its function protects against infection and regulates the inflammation response and autoimmunity [45]. The expression of the *CRP* gene showed a trend towards higher values in the laying hens housed in CC during the two sampling weeks evaluated. Previous studies have reported the overexpression of *CRP* under heat stress in poultry [46], stress from road transportation in Turkey [16], and transportation and heat shock stress in goats [35]. Even though in this study no significant differences were found, *CRP* expression is not investigated until now in laying hens housed in CC and CF systems, and to the best of our knowledge, the current study reported for the first time the changes in the *CRP* gene transcripts caused for the egg production system.

According to Sies [47], oxidative stress is the imbalance caused by a higher presence of oxidants compared to the presence of antioxidants that cause a disruption of redox signaling. During the synthesis of ATP, the respiratory chain produces reactive oxygen species (ROS) containing free radicals that produce damage to cellular structures, proteins, and lipids [19]. Various environmental stressors, such as SD, heat and cold stress, food restriction, and pollutants, impact the poultry industry [26]. Consequently, animals experiencing environmental stress exhibit elevated ROS levels, particularly in the liver, where the excessive ROS load overwhelms the buffering system, resulting in damage [19, 48]. NADPH oxidase 4 (NOX4), a membrane-bound complex, serves as an inflammatory stress protector and oxygen sensor. However, this complex generates O^2−^ (superoxide radicals) during NADP + biosynthesis, with its expression linked to increased ROS production under stress conditions [49–51]. The *NOX4* gene in laying hens of the CF system at 60 wk of production showed a higher expression, and this could be due to the litter facilities in this kind of production system that promote the air pollutants' circulation [51]. Primary pollutants in poultry houses, such as ammonia and dust, potentially compounded by fecal matter, bacteria, feathers, fungal spores, and straw, adversely affect the respiratory system and, at higher concentrations, the liver and kidneys [52, 53]. In mice, it has been probed that NOX proteins, including NOX4, are activated under a stimulus of dust extract [54]. However, at 80 wk of production, our results showed that the expression of the *NOX4* gene was higher in the laying hens housed in the CC group compared to the CF group; according to this, the hens could develop tolerance to the pollutants circulating as well as the results reported by Wu et al. [55].

In the oxidative stress, several antioxidant enzymes, such as superoxide dismutase (SOD), catalase (CAT), and glutathione peroxidase (GPX), participate in the dismutation of O_2_^−^ by SOD and transformation or reduction of hydrogen peroxide (H_2_O_2_) by CAT and GPX [56]. As previously mentioned, stress increases ROS levels inducing the upregulation of genes coding these enzymes as a control mechanism for cytotoxicity [57]. In the two weeks sampled (60 wk and 80 wk), *SOD1*, *CAT*, and *GPX1* genes were upregulated in the liver of hens of the CC group, and similar results were reported by Şimşek et al. [48] where the GSH serum level and CAT protein activity in ovarian tissues were found to be higher in the CC group than in the organic system. Furthermore, when comparing broiler chickens housed in CC and CF systems, CAT protein activity was notably higher during summer, while GSH serum levels peaked in autumn within the CC group [58]. On the other hand, comparable results were obtained in a study performed in broiler chickens exposed to heat stress that reported the upregulation of the *CAT* and *SOD*genes in the liver at five weeks of age and the expression of *SOD2* and *NOX4* at the 7-day post-hatch [51]. Additionally, the SOD protein activity was notably higher in broiler chicks of the CC systems, except for GPX serum levels, which remained unaffected [59]. On the contrary, in the case of the evaluation of SD, Simitzis et al. [60] and Hafez et al. [61] reported a lower activity of the GSH, SOD, GPX, and CAT in the high-density stocking. Consequently, our findings suggest that the upregulation of *SOD1*, *CAT*, and *GPX1* genes within the CC group may signify the activation of a protective mechanism aimed at mitigating liver damage caused by oxidative stress resulting from SD [51].

The avian liver serves as the primary site for *de novo* lipogenesis [19], a process crucial for synthesizing fatty acids essential in producing triacylglycerol and very low-density lipoprotein molecules. These molecules serve multiple purposes—they act as energy sources, integrate into cellular membranes, aid adipocyte differentiation, and participate in metabolic functions [62]. Moreover, in egg production, the liver plays a pivotal role by generating a specialized form of a very low-density lipoprotein, specifically aimed at transporting triglycerides to the oocyte [19]. Sterol regulatory element-binding proteins (SREBPs), as described by Bertolio et al. [63], are transcription factors that regulate the biosynthesis of lipids and adipogenesis. They exert control over the expression of genes responsible for synthesizing cholesterol, fatty acids, triacylglycerol, and phospholipids [63]. In the liver, the predominant isoform is *SREBF1* [64]. Our study evaluated the gene expression of *SREBF1* that was upregulated in the liver of hens housed in the CC group at 60 wk; however, at 80 wk, no statistical difference was found. Previously, in broiler chickens injected with dexamethasone (DEX), a glucocorticoid, *SREBF1*, showed a higher expression, which could influence the lipogenesis as well in the hepatic cells culture through the LXR-SREBP pathway [9, 12]. In hens housed in CC, the increment of the glucocorticoid level by stress may induce the upregulation of the *SREBF1* gene, as well as occurring in experimental animals and humans [65]. Similar results were found in broiler chickens exposed to heat stress where the *SREBF1* showed a higher expression compared to the control group [20, 66]. The upregulation of *SREBF1* suggests an increase in the rate of fat synthesis due to its expression stimulated by insulin and glucose levels that increased due to the elevated levels of glucocorticoids such as CORT [65, 67]. These results could indicate a fat accumulation induced by *SREBF1*, potentially leading to metabolic disorders due to excessive fat deposition and compromised transport of triglycerides, attributed to the downregulation of genes like *apoB* [68]. Furthermore, another gene evaluated was *FXR* (farnesoid X receptor), which is a nuclear receptor whose function is to be a sensor of bile acids, regulating its production, transport, and conjugation [12, 69]. While our study did not reveal significant differences in *FXR* expression, Hu et al. [12] and Yin et al. [20] reported higher *FXR* expression levels among bird groups subjected to DEX and heat stress, respectively.

## 5. Conclusion

Hens housed in CC showed upregulation of *SOD1*, *CAT*, and *GPX1* genes within the liver, a pattern associated with oxidative stress. Furthermore, the CC system may induce changes in the lipid synthesis which in the long term could affect the hen's performance based on the modulation of the *SREBF1* gene. *SOD1*, *CAT*, and *GPX1* genes can be used as biomarker candidates for oxidative stress in laying hens in welfare studies. However, further research is necessary to determine the influence of the production system on stress, oxidative stress, and lipid metabolism using complementary techniques.

## Figures and Tables

**Table 1:** Sequences of primers used for the gene expression of the interest genes.

Genes			Primer sequence 5′-3′	Amplicon size (pb)	References
Acute phase proteins	*AGP*	F	TGGGTGTACATCATGGGTGC	80	Authors
		R	AGGGTGAAGGTCGCGTACTT		
	*CRP*	F	ATACGTCGCCTTCCACATCC	150	
		R	TCGTTGCCCACCACGTA		
Oxidative stress	*NOX4*	F	TGTACCGCTACATCCGCAG	159	
		R	GGCTTTCCAGTCCAGACACT		
	*SOD1*	F	CGGGCCAGTAAAGGTTACTGGAA	83	
		R	TGTTGTCTCCAAATTCATGCACATG		
	*CAT*	F	TCGTCTCTTTGCGTATCCTGA	80	
		R	TGTAGGGGCAATTCACAGGA		
	*GPX1*	F	CAACGGCTTCAAACCCAACT	159	
		R	ACCGGCGACCAGATGATGTA		

Lipid metabolism	*SREBF1*	F	GCCCTCTGTGCCTTTGTCTTC	130	Hu et al. [12]
		R	ACTCAGCCATGATGCTTCTTCC		
Bile acid receptor	*FXR*	F	AGTAGAAGCCATGTTCCTCCGTT	182	
		R	GCAGTGCATATTCCTCCTGTGTC		

Actin beta	*ACTB*	F	GCCCCCAAAGTTCTACAAT	110	Rodríguez‐Hernández et al. [22]
		R	AGGCGAGTAACTTCCTGTA		

## Data Availability

The data were obtained from the study. Also, all the datasets generated or analyzed during this study are included within the article.
